# Unripe *Carica papaya* Protects Methylglyoxal-Invoked Endothelial Cell Inflammation and Apoptosis via the Suppression of Oxidative Stress and Akt/MAPK/NF-κB Signals

**DOI:** 10.3390/antiox10081158

**Published:** 2021-07-21

**Authors:** Wattanased Jarisarapurin, Khwandow Kunchana, Linda Chularojmontri, Suvara K. Wattanapitayakul

**Affiliations:** 1 Department of Pharmacology, Faculty of Medicine, Srinakharinwirot University, Bangkok 10110, Thailand; wattanased.jarisalapurin@g.swu.ac.th (W.J.); Khwandow.kun@g.swu.ac.th (K.K.); 2 Department of Preclinical Sciences, Faculty of Medicine, Thammasat University, Pathum Thani 12121, Thailand; clinda@staff.tu.ac.th

**Keywords:** methylglyoxal, dicarbonyl stress, inflammation, apoptosis, advanced glycation end products (AGEs), antioxidants, papaya, diabetes, vascular complications

## Abstract

Methylglyoxal (MGO), a highly reactive dicarbonyl compound, causes endothelial oxidative stress and vascular complications in diabetes. Excessive MGO-induced ROS production triggers eNOS uncoupling, inflammatory responses, and cell death signaling cascades. Our previous study reported that unripe *Carica papaya* (UCP) had antioxidant activities that prevented H_2_O_2_-induced endothelial cell death. Therefore, this study investigated the preventive effect of UCP on MGO-induced endothelial cell damage, inflammation, and apoptosis. The human endothelial cell line (EA.hy926) was pretreated with UCP for 24 h, followed by MGO-induced dicarbonyl stress. Treated cells were evaluated for intracellular ROS/O_2_^•−^ formation, cell viability, apoptosis, NO releases, and cell signaling through eNOS, iNOS, COX-2, NF-κB, Akt, MAPK (JNK and p38), and AMPK/SIRT1 autophagy pathways. UCP reduced oxidative stress and diminished phosphorylation of Akt, stress-activated MAPK, leading to the decreases in NF-kB-activated iNOS and COX-2 expression. However, UCP had no impact on the autophagy pathway (AMPK and SIRT1). Although UCP pretreatment decreased eNOS phosphorylation, the amount of NO production was not altered. The signaling of eNOS and NO production were decreased after MGO incubation, but these effects were unaffected by UCP pretreatment. In summary, UCP protected endothelial cells against carbonyl stress by the mechanisms related to ROS/O_2_^•−^ scavenging activities, suppression of inflammatory signaling, and inhibition of JNK/p38/apoptosis pathway. Thus, UCP shows considerable promise for developing novel functional food and nutraceutical products to reduce risks of endothelial inflammation and vascular complications in diabetes.

## 1. Introduction

Glycolytic overload has contributed to the development and comorbidity of numerous metabolic syndromes and chronic disorders, including diabetic complications, cardiovascular disease, and cancer [1]. Methylglyoxal (MGO) is a by-product generated during the glycolysis pathway, as well as being present in the natural resources such as honey, coffee, wine, whisky, and bread [2]. MGO causes dicarbonyl stress and inflammation by altering the protein and DNA structure, resulting in the formation of irreversible advanced glycation end products (AGEs) [3]. MGO and MGO-derived AGEs induce mitochondrial dysfunction, endoplasmic reticulum (ER) stress, activation of NADPH oxidase, leading to the overproduction of reactive oxygen species (ROS) [4]. Oxidative stress stimulates the mitogen-activated protein kinase (MAPK) pathway to send the downstream signals to ERK, p38, and JNK, causing nuclear factor kappa B (NF-κB) translocation. As NF-κB is activated and enters the nucleus, it regulates the transcription of the pro-inflammatory mediators cyclooxygenase-2 (COX-2) and inducible nitric oxide synthase (iNOS), which promote endothelial cell inflammation and apoptosis, invoking endothelial dysfunction and diabetic vascular complications [5,6]. Additionally, AGEs activate the phosphatidylinositol 3 kinase (PI3K)/protein kinase B (Akt) signaling pathway that connects with the network of NF-κB-regulated cell survival, inflammation, and apoptosis [7]. Recent study has shown that the impaired autophagy in diabetic nephropathy also promotes apoptosis, inflammation, and cellular injury responses, causing vascular complications which are regulated by the upstream autophagic signaling cascades, including AMP-activated protein kinase (AMPK) and sirtulins (SIRTs) [8].

Endothelial nitric oxide synthase (eNOS) is the key regulator of NO synthesis. NO initiates vasodilation through an activation of soluble guanylyl cyclase to generate cyclic guanosine monophosphate (cGMP) which sets off several molecular events, such as reducing intracellular Ca^2+^ concentration, and subsequently causing vascular smooth muscle relaxation. Under dicarbonyl stress conditions, the overproduced ROS oxidize the eNOS cofactor tetrahydrobiopterin (BH_4_), resulting in O_2_^•−^ formation. This highly reactive radical directly destroys NO, and transforms it to peroxynitrite (ONOO^−^), a reactive molecule that gives rise to uncoupled eNOS, diminished NO bioavailability, and endothelial dysfunction [9,10]. As a consequence, the dicarbonyl stress in diabetes is strongly associated with vascular abnormality and the development of aging-related complications [11]. Given all the detrimental effects of oxidative stress in diabetes, the use of antioxidants is on the horizon of research prospects to defer disease complications. A cohort study by Mancini et al. [12] enrolled 1751 women with type 2 diabetes and followed over 15 years. It indicates significant inverse association between the dietary total antioxidant capacity and the risk of type 2 diabetes. Natural antioxidants, targeting endothelial cell oxidative stress caused by MGO, are excellent candidates for the prevention of vascular complications in diabetes and delay aging-related tissue dysfunction. For example, the polyphenol phytoantioxidant called phloretin, obtained from apples, can inhibit MGO-induced AGEs formation in human endothelial cells by modifying RAGE/p38 MAPK/NF-κB signaling pathway [13]. The phenolic compound catechins, commonly found in foods and beverages such as berries, tea, and cocoa, inhibits MGO-induced mitochondrial damage and apoptosis in human endothelial cells [14].

Functional foods and several herbs have high potential to be used as adjunct therapy for diabetes [15]. Thus, it is essential to provide scientific data to promote the incorporation of functional foods into daily diets for the strengthening of endogenous antioxidant defenses against oxidative damage caused by AGEs. The tropical fruit *Carica papaya* (papaya or pawpaw) can be found in several regions, including southeast Asia, south America, and Mexico. Papaya is usually consumed in a ripe stage and has been the subject of several studies for its antioxidant benefits. However, many countries in southeast Asia, such as Thailand, unripe *Carica papaya* (UCP) is used as the main ingredient in a famous dish known as Thai green papaya salad, or Som Tam. The green papaya fruits contain several phytoantioxidants, including vitamin C, quercetin, kaempferol, caffeic acid, chlorogenic acid, and enzymes papain and carpaine [16]. Research on green papaya fruits reveal a wide range of pharmacological effects such as antioxidants, anti-inflammation [17], anti-aging [18], and wound healing [19], but the prevention of endothelial cell damage in dicarbonyl stress mimicking diabetic condition has not been reported. Our previous study shows that UCP protected against H_2_O_2_-induced endothelial cell death, due to its strong antioxidant properties, as well as the specific scavenging activity to eliminate H_2_O_2_, O_2_^•−^, OH, and HOCl [20]. Therefore, this study further investigated the cytoprotective effects of UCP against MGO-induced endothelial cell oxidative stress, inflammation, and apoptosis.

## 2. Materials and Methods

### 2.1. Chemical and Cell Culture Protocol

Cell culture and analytical grade chemicals in this study were obtained from MilliporeSigma (Burlington, MA, USA) unless otherwise stated. The human endothelial cell line, EA hy926 (CRL-292™) was purchased from ATCC^®^ (Manassas, VA, USA) to perform the dicarbonyl-induced oxidative stress model in cell culture. Dulbecco’s Modified Eagle Medium (DMEM) supplemented with 10% fetal bovine serum (FBS), 100 U/mL penicillin, and 100 µg/mL streptomycin (Thermo Fisher Scientific, Waltham, MA, USA) was used to culture the endothelial cell in the autoflow NU-4850 humidity control water-jacket laboratory CO_2_ incubator (NuAire, Plymouth, MN, USA) maintained under a humidified 5% CO_2_ chamber and incubated at 37 °C. The culture medium was changed every 3 days and subcultured when cells were grown to 80% confluence. In each experiment, cells were seeded in culture plates and incubated for 18–24 h. Cells were pretreated with fresh media containing UCP at a concentration of 10, 100, and 1000 µg/mL for 24 h. Then, cells were incubated with MGO at the concentration of 800 µM (#M0252, MilliporeSigma, Burlington, MA, USA) for 24 h. After incubation, cells were harvested for further evaluations by each detection method. MGO was prepared to 1 M stock with type 1 sterile ultrapure water (Milli-Q^®^ type 1 ultrapure water systems, MilliporeSigma, Burlington, MA, USA) and then diluted to the desired concentrations.

### 2.2. Unripe Carica papaya (UCP) Fruit Juice Preparation

The preparation of fruit juice powder was described in our previous study which obtained at the yield of 1.18% (w/w) [20]. Briefly, cleaned UCP fleshes were sliced into small pieces and extracted via juice extractor (Braun MP75 Multipress Compact, Braun AG, Kronberg, Germany). The fresh juice was kept on ice during filtering through sterile qualitative paper (Whatman^®^ grade 1 filter paper). The fruit extract was then lyophilized to dry powders and stored at −40 °C until use. The UCP stock solutions were freshly prepared for each experiment by dissolving the powder in type 1 sterile ultrapure water (Milli-Q^®^ type 1 ultrapure water systems, MilliporeSigma, Burlington, MA, USA).

### 2.3. Cell Viability Assay

The preventive effect of UCP on MGO-induced EA hy926 cell death was determined by 3-(4,5-Dimethylthiazol-2-yl)-2,5-Diphenyltetrazolium Bromide (MTT) cell viability assay (Bio Basic, Amherst, NY, USA). Briefly, 1 × 10^4^ cells were seeded in a 96-well cell culture plate for 18–24 h. UCP was pretreated on endothelial cells for 24 h before challenging with MGO. For the detection of cell viability, DMEM containing 0.25 mg/mL MTT was added to the cell culture and incubated for 3 h. The absorbance of DMSO-dissolved formazan was measured at the wavelength 550 nm using SpectraMax^®^ M2e microplate reader (Molecular Devices, San Jose, CA, USA). Data are demonstrated as the percentage of cell viability compared with the vehicle-treated group (100%).

### 2.4. Measurement of Apoptotic Cells

The Guava^®^ Nexin Reagent (Luminex Corporation, Austin, TX, USA) containing Annexin V-PE and 7-AAD was used to determine cell apoptosis. Cells were seeded in a 35-mm cell culture plates at 1.5 × 10^5^ cells/dish and cultured for 18–24 h. After treatment with various concentrations of UCP (10, 100, and 1000 µg/mL) and 800 µM MGO, cells were collected by trypsinization and resuspended in fresh media containing 1% FBS. Cell concentration was adjusted to 0.2–1 × 10^6^ cells/mL. One hundred microliters of each sample were mixed with Nexin Reagent and incubated in the dark at room temperature for 20 min. Acquired samples were determined for apoptotic cells using a flow cytometer cooperative with Guava Nexin analysis software (Luminex Corporation, Austin, TX, USA). Data are presented as percentage of apoptotic cells.

### 2.5. NO Release

The release of NO from the endothelial cells treated with UCP and MGO was determined by Griess assay as described by Bryan and Grisham [21]. Briefly, cells were seeded in a 6-well cell culture plate at the concentration of 5 × 10^5^ cells/well. After treatment, the medium from each sample was collected by centrifugation at 2000× *g* for 1 min. Then, the samples were incubated for 30 min with NADPH and nitrate reductase at the final concentration of 55 µg/mL and 150 mU/mL, respectively. One hundred microliters of each sample or standard were mixed with 100 µL Griess reagent containing 1% sulfanilamide dissolved in 3 M HCl and 0.1% N-(1-naphthyl) ethylenediamine dihydrochloride (NED) dissolved in distilled water. After 15 min, the reaction mixtures were measured for the absorbance at the wavelength 540 nm, using a microplate reader (Molecular Devices, San Jose, CA, USA). Data are presented as the units of NaNO_2_ concentration in µM.

### 2.6. Measurement of Intracellular ROS

The effect of UCP on MGO-induced EA hy926 cell oxidative stress was determined by 2′,7′-Dichlorofluorescin diacetate (DCFH-DA) ROS probe and detected by flow cytometry. Cells were seeded in a 35-mm cell culture dish at the concentration of 1.5 × 10^5^ cells/dish. After treatment, cells were washed twice with 1X PBS and incubated with the medium containing 25 µg/mL DCFH-DA for 30 min. Cells were collected by trypsinization followed by two-time washing with 1X cold DPBS. The cell concentration was adjusted to 500 cells/µL. The fluorescent intensity, representing the intracellular ROS, was determined by AMNIS^®^ ImageStreamX Mark II imaging (Luminex Corporation, Austin, TX, USA) and Guava^®^ easyCyte^™^ 8 HT flow cytometer (Luminex Corporation, Austin, TX, USA). The ROS baseline range was evaluated referring to unstained cell populations. The intracellular ROS was presented as the percentage of mean fluorescence intensity compared with the vehicle-treated group.

### 2.7. Measurement of Intracellular Superoxide

The amount of Intracellular superoxide was determined by the specific probe dihydroethidium or DHE (Thermo Fisher Scientific, Waltham, MA, USA). Cells were seeded in a 35-mm cell culture dish at 1.5 × 10^5^ cells/dish and incubated for 18–24 h. After treatment, cells were washed with 1X PBS and double stained with 10 µM DHE and Hoechst for 30 min. Cells were collected by trypsinization followed by 1X cold PBS washes. The cell concentration was adjusted to 500 cells/µL using 1X cold DPBS. The percentage of mean fluorescence intensity of intracellular superoxide was determined by a flow cytometer, in which an unstained cell population was used to set the superoxide baseline range. The proportions of mean fluorescence intensity were used to compare among the intracellular superoxide levels using vehicle-treated group as a reference. The endothelial cells cultured on Nunc™ Lab-Tek™ chamber system (Thermo Fisher Scientific, Waltham, MA, USA) were observed under the Olympus FluoView FV10i-confocal laser scanning microscope (Olympus Corporation, Shinjuku, Tokyo, Japan).

### 2.8. Western Blot Analysis

The whole cell lysates and nuclear lysates were collected using RIPA buffer and nuclear extraction kit (Cayman Chemical, Ann Arbor, MI, USA), respectively. The protein concentrations of each sample were determined by Bio-Rad protein assay (Bio-Rad Laboratories Ltd., Hercules, CA, USA). An equal amount of each protein sample was separated by the SDS-PAGE, and then transferred to Amersham Hybond^™^ P 0.45 PVDF blotting membrane (GE Healthcare, Chicago, IL, USA) using the Mini-PROTEAN Tetra system (Bio-Rad Laboratories Ltd., Hercules, CA, USA). The blotted membranes were soaked in the blocking solution (5% BSA or non-fat dry) in Tris-buffered saline with 0.1% Tween^®^ 20 detergent (TBST) for 1 h. Then, the membranes were incubated overnight with the primary antibodies at the dilution 1:1000, including COX-2 (#12282), NF-κB (#4764), β-actin (#3700), p-Akt (#4051), Akt (#9272), p-JNK (#4668), JNK (#9252), p-p38 (#9215), p38 (#8690), p-eNOS (#9570), p-AMPKα (#2535), AMPKα (#2532), p-SIRT1 (#2314), SIRT1 (#9475) (Cell Signaling Technology, Danvers, MA, USA), iNOS (#sc-7271), or eNOS (#sc-136977) (Santa Cruz Biotechnology, Dallas, TX, USA). After incubation, the blotted membranes were washed with 1X TBST three times and incubated for 1 h with 1:3000 anti-rabbit (#7076) or anti-mouse (#7074) IgG linked with HRP (Cell Signaling Technology, Danvers, MA, USA). After several washes, the specific protein bands on the membranes were detected with Amersham ECL Select Western blot reagent (GE Healthcare, Chicago, IL, USA). The photos of membranes were collected for analysis by a gel documentation system (UVITEC, Cambridge, UK). The Image J software version 1.52a (https://imagej.net/ImageJ1) was used to quantify and analyze the appeared protein bands.

### 2.9. Statistical Analysis

Data are represented as mean ± SEM calculated from at least three separate experiments. One-way analysis of variance (ANOVA) was used for the comparison of all pairs of samples while *t*-test was applied to compare two groups of samples. The statistical significance was set at the *p* values less than 0.05.

## 3. Results

### 3.1. Effects of UCP on MGO-Induced Endothelial Cell Death

**Figure 1:**
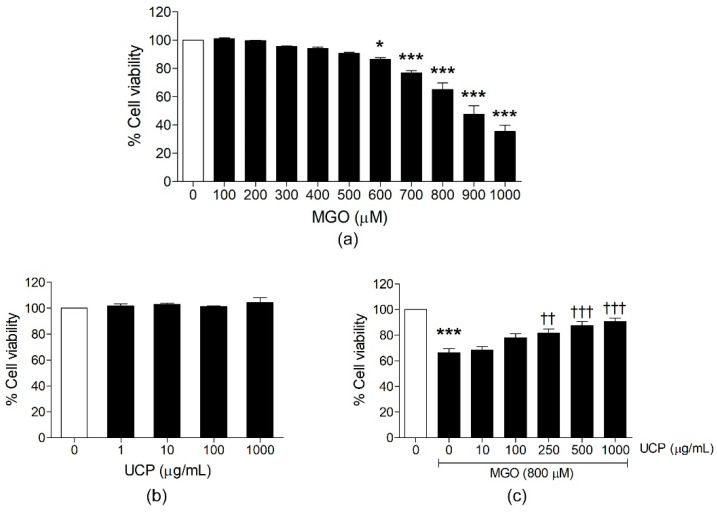
The protective effect of UCP on MGO-induced EA hy926 cell death. MTT assay was used to determine cell survival as described in Materials and Methods. (**a**) Effect of various concentrations MGO (100–1000 µM) on cell viability. (**b**) Effects of UCP (10–1000 µg/mL) on cell viability. (**c**) Preventive effect of UCP pretreatment on MGO-decreased cell viability. Data are presented as mean ± SEM (n ≥ 3). *, *p* < 0.05 and ***, *p* < 0.001 when compared with the vehicle-treated group; ††: *p* < 0.01; and †††: *p* < 0.001 when compared with 800 µM MGO challenged group.

MTT cell viability assay showed that endothelial cell challenged with MGO at the concentrations lower than 600 µM had no significant toxicity, while MGO at concentrations 600, 700, 800, 900, and 1000 µM significantly decreased percent cell viability to 86.59 ± 1.09, 76.95 ± 1.55, 65.14 ± 4.69, 47.77 ± 5.93, and 35.48 ± 4.46%, respectively, when compared with the vehicle-treated group (Figure 1a). Among the five toxic concentrations, MGO at 800 µM was selected for further experiments in this study as it moderately induced cell death (around 30–40%) to the degree that the cytoprotection by UCP could be clearly observed. Endothelial cells preincubated with UCP (1, 10, 100, and 1000 µg/mL) for 24 h had no toxic effect on EA hy926 cells (Figure 1b). UCP pretreatment (250, 500, and 1000 µg/mL) prior to MGO incubation significantly improved cell viability to 81.68 ± 3.00, 87.65 ± 3.21, and 90.60 ± 2.75%, respectively, when compared with MGO treated group (*p* < 0.01) (Figure 1c).

### 3.2. Effects of UCP on MGO-Induced Cell Apoptosis

**Figure 2:**
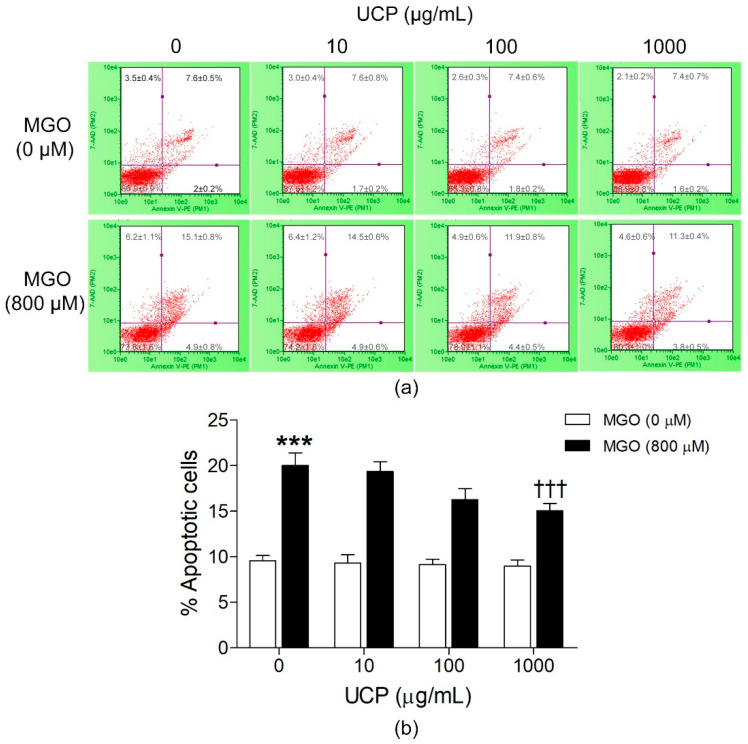
Effects of UCP on MGO-induced cell apoptosis. Apoptotic cells were determined by Annexin V-PE and 7-AAD probe and analyzed by a flow cytometer. (**a**) Dot plot diagrams obtained from flow cytometry. (**b**) A graphic representation of calculated percent apoptosis of cell populations in each treatment group. Data are presented as mean ± SEM. ***, *p* < 0.001 when compared with the vehicle-treated group; †††, *p* < 0.001 when compared with MGO treated group.

The preventive effect of UCP on cell apoptosis was evaluated by Guava^®^ Nexin assay designed to use with flow cytometry system (Figure 2). Under the basal condition, EA hy926 cells entered apoptotic stage at approximately 10%. UCP treatment did not change the percent apoptosis in this condition. However, endothelial cells preincubated with UCP dose-dependently reduced MGO-induced apoptosis, but it reached statistical significance at the concentration of 1000 µg/mL. The percent apoptotic cells were lowered to 15.08 ± 0.76% (*p* < 0.05) when compared with the MGO challenged group (20.04 ± 1.38%).

### 3.3. Effects of UCP on Intracellular ROS Levels

**Figure 3:**
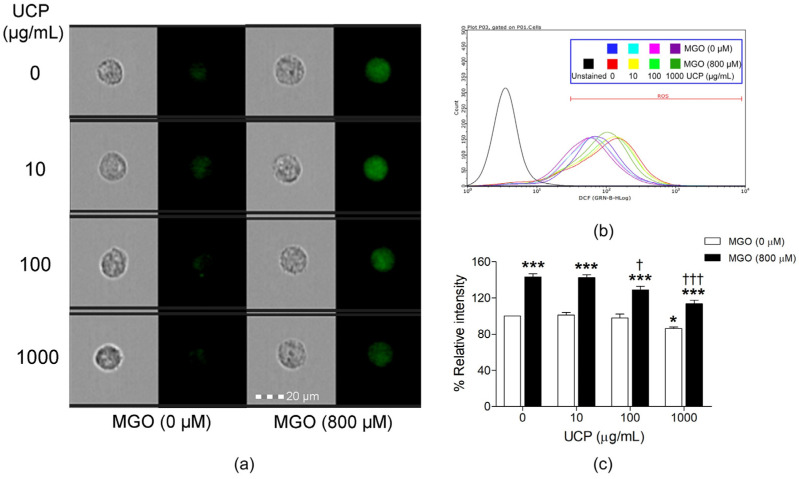
Effects of UCP pre-incubation on MGO-induced intracellular ROS production. (**a**) The representative photos of DCFH-DA green fluorescence probe detecting intracellular ROS in a single-cell configuration analyzed by imaging flow cytometer. (**b**) Flow cytometry histogram of relative intracellular ROS levels in each sample group. (**c**) A graphical representation of percent relative fluorescent intensity of cell populations in each treatment group. Data are presented as mean ± SEM of n ≥ 3. *, *p* <0.05; and ***, *p* < 0.001, when compared with the vehicle-treated group; †, *p* < 0.05; and †††, *p* < 0.001, when compared with MGO-treated group.

The inhibitory effect of UCP on Intracellular ROS production was determined by DCFH-DA staining. UCP concentration-dependently reduced the intracellular fluorescence intensities as analyzed under the single-cell imaging flow cytometer (Figure 3a) and depicted in the flow cytometry histogram (Figure 3b). Only cells treated with UCP (1000 µg/mL) significantly decreased intracellular ROS to 86.10 ± 1.74% (*p* < 0.05) when compared with the vehicle-treated group (100%). By contrast, MGO treatment significantly increased intracellular ROS up to 143.23 ± 3.26% (*p* < 0.001). UCP pretreatment at concentrations of 100 and 1000 µg/mL prevented MGO-induced oxidative stress by significantly decreasing intracellular ROS to 128.87 ± 4.02% and 113.29 ± 4.10%, respectively, when compared with MGO-treated group (Figure 3c).

### 3.4. Effects of UCP on Intracellular Superoxide Levels

**Figure 4:**
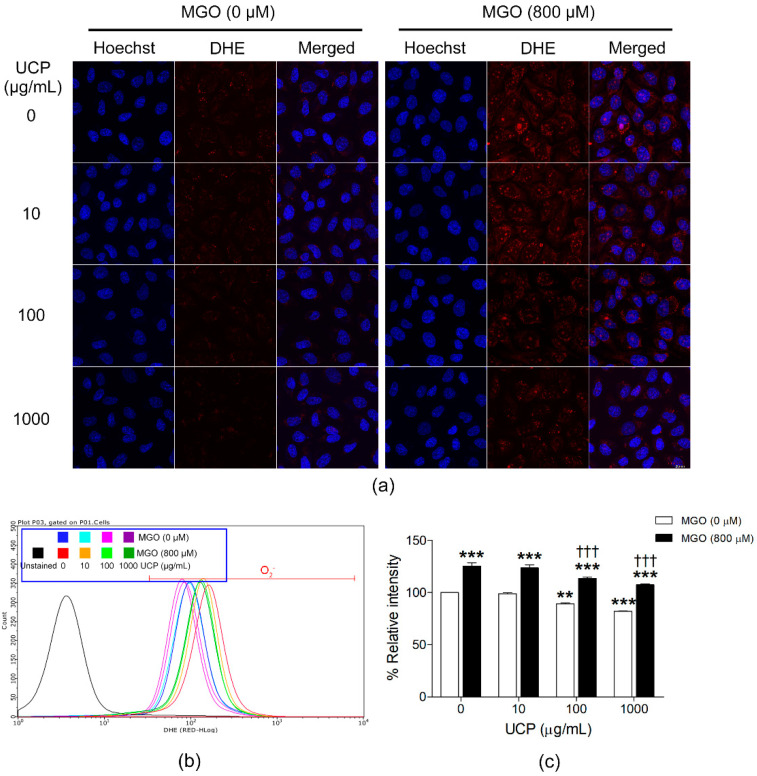
Effects of UCP on MGO-induced intracellular superoxide production. (**a**) The representative photos of DHE fluorescence probed detecting a single-cell intracellular superoxide that observed under a fluorescent microscope. (**b**) Flow cytometry histogram of each sample group. (**c**) A graph of calculated percent relative intensity of each sample group (**b**). Data are presented as mean ± SEM. **, *p* <0.01; and ***, *p*, < 0.001, when compared with vehicle-treated group; †††, *p* < 0.001, when compared with MGO exposed group.

Intracellular O_2_^•−^ levels were determined by DHE staining probe. The fluorescence signal was visualized under the confocal laser microscope, and the relative O_2_^•−^ levels were determined by flow cytometer (Figure 4a,b). UCP decreased O_2_^•−^ in EA.hy926 cells both in the presence and absence of MGO. UCP at the concentrations 100 and 1000 µg/mL significantly decreased the relative amount of O_2_^•−^ to 88.91 ± 1.13% and 81.81 ± 0.39% at *p* < 0.01 and *p* < 0.001, respectively. By contrast, MGO significantly increased intracellular O_2_^•−^ to 125.48 ± 2.98% when compared with the vehicle-treated group. Cells incubated with UCP (100 and 1000 µg/mL) prior to MGO challenge showed significant decreases in percent mean intensities of DHE fluorescence to 113.46 ± 1.05% and 107.34 ± 0.80%, respectively (*p* < 0.001) (Figure 4c).

### 3.5. Effects of UCP on eNOS and NO Release

**Figure 5:**
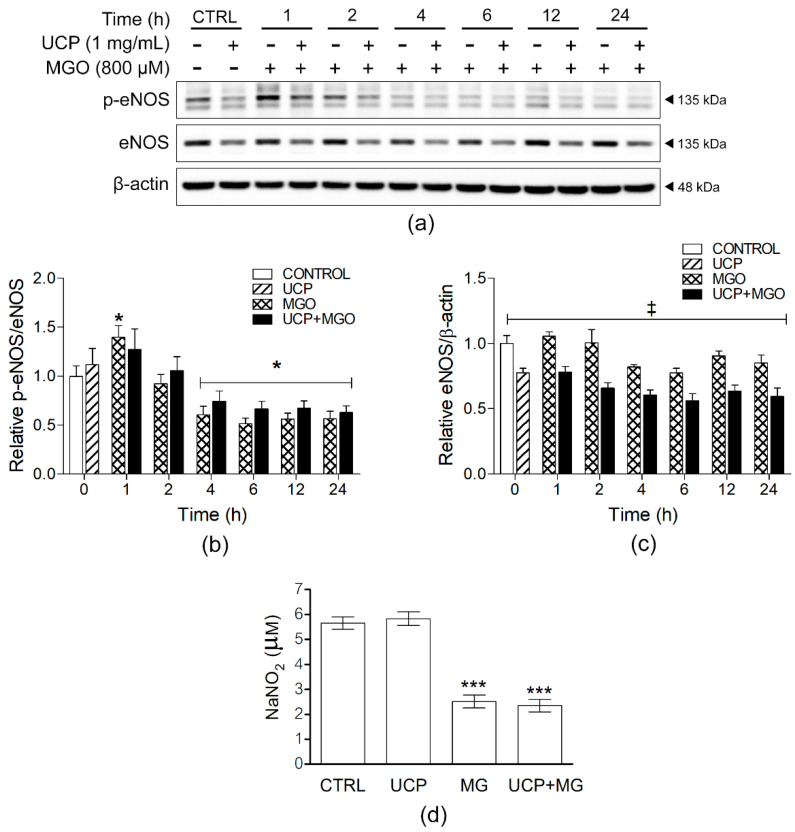
Effects of UCP pretreatment on eNOS phosphorylation and nitric oxide levels in EA hy926 cell exposed MGO. (**a**) Representative Western blot protein bands of cell lysates from each sample group. (**b**) Relative ratios of p-eNOS/eNOS and (**c**) p-eNOS/β-actin. (**d**) NO releasing levels from cells in each treatment group. Data are presented as mean ± SEM. *, *p* < 0.05; ***, *p* < 0.001, when compared with vehicle-treated group; ‡, *p* < 0.05, when compared MGO-exposed group at the same time point.

The effect of UCP on eNOS protein phosphorylation (p-eNOS/eNOS) and NO release was evaluated by Western blot and Griess assay, respectively (Figure 5 and Figure S1). The time-course study of eNOS phosphorylation showed that MGO activated eNOS phosphorylation (1.4 ± 0.12 folds) at as early as 1 h but subsided at 4 h (0.61 ± 0.09 fold) and afterward. These effects of MGO were not altered by UCP pre-incubation (Figure 5a,b). Interestingly, UCP significantly decreased eNOS existence (eNOS/β-actin) down to 0.77 ± 0.04 fold and maintained this approximate level at all incubation time points; however, MGO did not significantly change eNOS/β-actin ratio (Figure 5c). For the assay of NO release, cells incubated with UCP alone did not have any impact on the release of NO in the culture media. On the contrary, MGO notably decreased NO release down to 0.44 ± 0.10 fold, and cells preincubated with UCP did not have any significant alteration of NO release (Figure 5d).

### 3.6. Effects of UCP on iNOS, COX-2, and NF-κB in MGO-Induced Cell Inflammation

**Figure 6:**
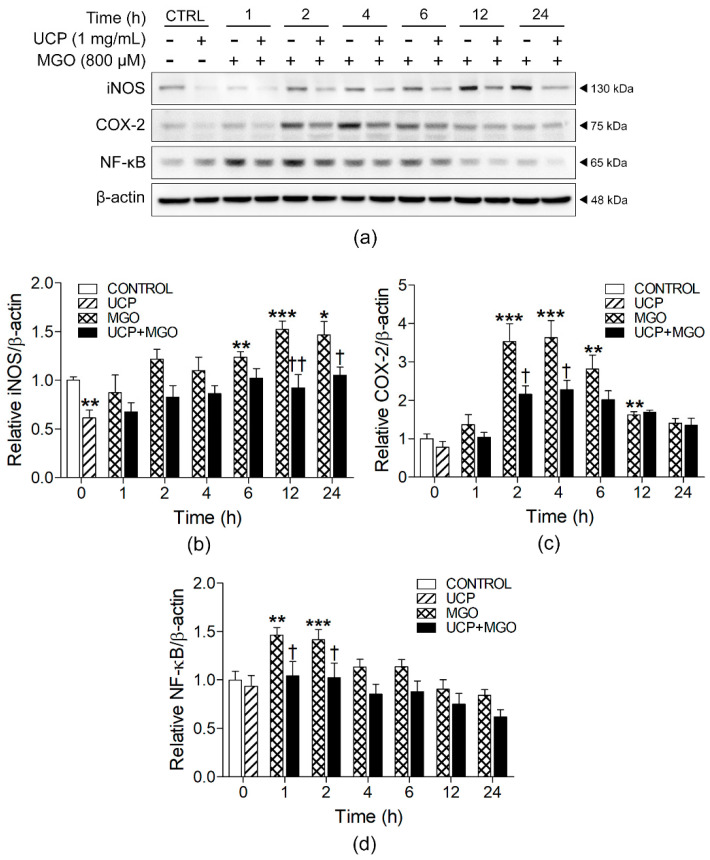
Effects of UCP pretreatment on MGO-induced cell inflammatory signaling protein expression. (**a**) Representative Western blot protein bands. (**b**) Relative ratios of iNOS/β-actin and (**c**) COX-2/β-actin. (**d**) The ratios of NF-κB/β-actin. Data are presented as mean ± SEM. *, *p* < 0.05; **, *p* < 0.01; and ***, *p* < 0.001, when compared with each treated group; †, *p* < 0.05; and ††, *p* < 0.01, when compared with MGO challenged group at the same time point.

The Western blot bands of whole cell and nuclear protein lysates of each treatment were analyzed for the activation of inflammatory signaling protein consisting of COX-2, iNOS, and NF-κB (Figure 6a and Figure S2). The MGO challenged cells showed significant activation of iNOS, which occurred at 6 h (1.24 ± 0.06 folds) and increasingly elevated until 24 h (1.5 folds) after incubation (*p* < 0.05) (Figure 6b). Likewise, COX-2 expression was significantly enhanced within 2 h (3.53 ± 0.46 folds) (*p* < 0.05) until receding to approximately at the basal level after 24-h incubation time (Figure 6c). The translocation of NF-κB transcription factor into the nuclei was significantly elevated from 1 h (1.5 folds) (*p* < 0.05) until leveled down to the baseline at 4 h after MGO incubation, when compared with the vehicle-treated group (Figure 6d). UCP pretreatment significantly decreased iNOS/β-actin ratios at 12 and 24 h (0.92 ± 0.13 and 1.05 ± 0.09 folds, respectively) when compared MGO challenged group. Similarly, COX-2 expression was also significantly reduced at 2 and 4 h (2.16 ± 0.21 and 2.27 ± 0.25 folds, respectively) whereas NF-κB translocation were completely suppressed at the beginning of their rises at 1 and 2 h (1.47 ± 0.08 and 1.42 ± 0.10 folds, respectively) after MGO treatment (Figure 6b–d).

### 3.7. Effect of UCP on the Signaling of Akt, JNK, and p38 in MGO-Induced Cell Survival and Cell Death

**Figure 7:**
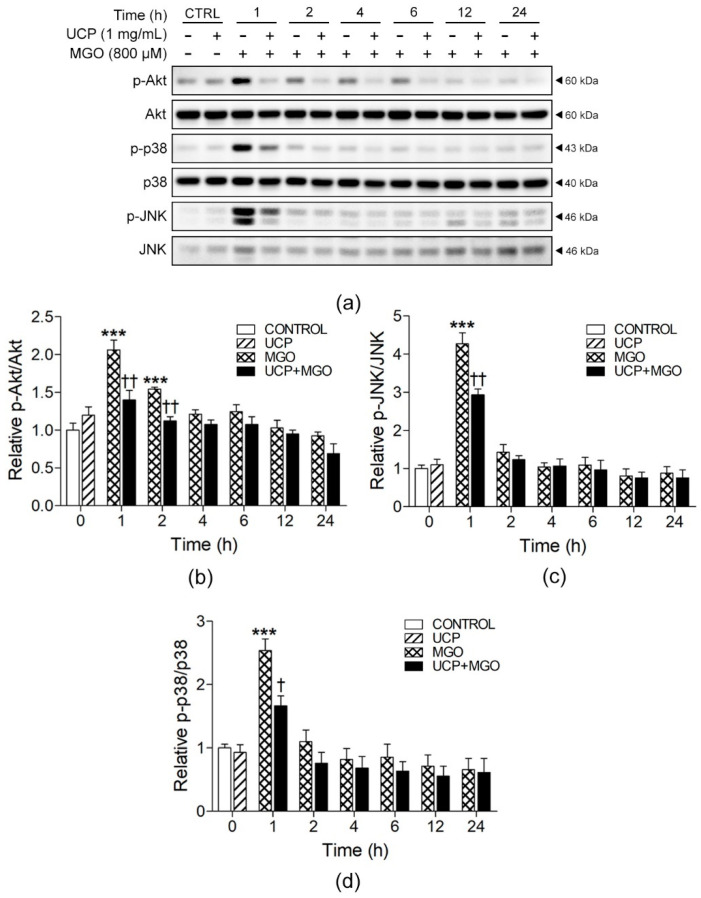
Effects of UCP pre-treatment on MGO-induced cell survival and cell death pathways. (**a**) Representative Western blot protein bands. Relative ratios of (**b**) p-Akt/Akt, (**c**) p-JNK/JNK, and (**d**) p-p38/p38. Data are presented as mean ± SEM. ***, *p* < 0.001, when compared with each treated group; †, *p* < 0.05 and ††, *p* < 0.01, when compared MGO-exposed group at the same time point.

Figure 7 and Figure S3 present Western blot analyses of signaling phosphorylation of Akt, JNK, and p38. Endothelial cells exposed to MGO showed significant increases in Akt phosphorylation within 1 h (2.06 ± 0.13 folds) and decreased afterward to the basal level within 24 h (Figure 7b). UCP pre-treatment significantly inhibited the early activations of Akt phosphorylation at 1 and 2 h where the ratios of p-Akt/Akt were down to 1.40 ± 0.12 and 1.12 ± 0.06 folds, respectively. Correspondingly, the phosphorylation of JNK and p38 also significantly increased at the same time point (2.53 ± 0.18, and 4.27 ± 0.29 folds, respectively) and diminished right after the first hour of activation (Figure 7c,d). Similarly, within one hour of MGO incubation, UCP markedly lessened the MGO-activated phosphorylations of p38 and JNK down to 2.93 ± 0.15 and 1.66 ± 0.16 folds, respectively.

### 3.8. Effects of UCP on the Signaling of AMPKα and SIRT1 in MGO-Induced Autophagy

**Figure 8:**
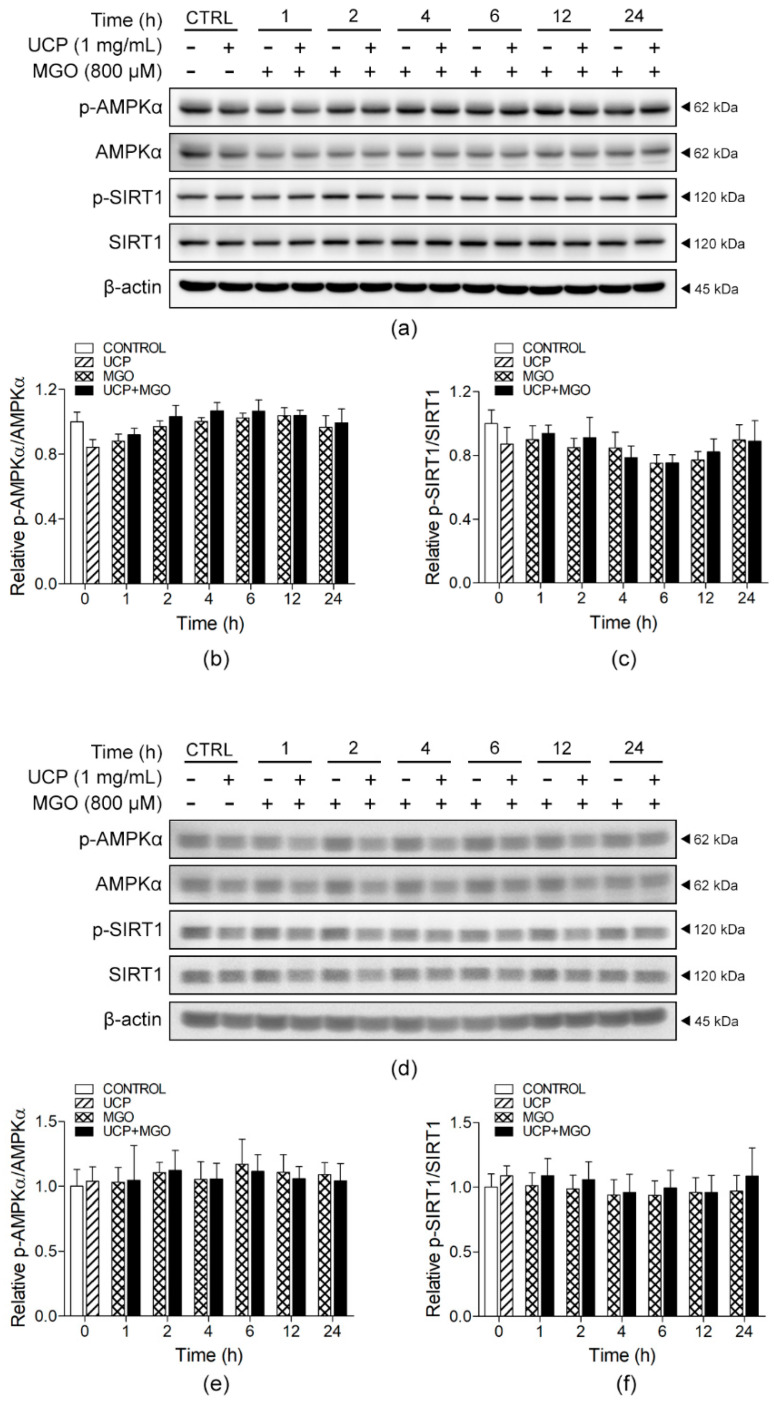
Effects of UCP pretreated on MGO-induced autophagy in high and low glucose DMEM media. (**a**) Representative Western blot protein bands of cell lysates obtained from high glucose culture showing (**b**) p-AMPKα/AMPKα ratios and (**c**) p-SIRT1/SIRT1 ratios. (**d**) Representative Western blot protein bands of cell lysates obtained from low glucose culture showing (**e**) p-AMPKα/AMPKα ratios and (**f**) p-SIRT1/SIRT1 ratios. Data are presented as mean ± SEM.

Shown in Figure 8 and Figure S4 are the Western blot analyses assessing the effects of UCP on MGO-induced endothelial cell autophagy under high and low glucose conditions. During the 0–24 h time-course study, MGO had no influence on the autophagic signaling through AMPKα/SIRT1. Likewise, UCP pre-incubation did not affect the signaling of AMPKα and SIRT1 in both conditions.

## 4. Discussion

Glycolytic overload causes an extreme accumulation of the highly reactive dicarbonyl compound, resulting in a harmful condition called dicarbonyl stress [22]. MGO is the most relevant reactive dicarbonyl substance in diabetic conditions that triggers non-enzymatic glycation, leading to the irreversible overproduction of AGEs [11]. MGO mechanistically induces ROS production and oxidative stress in endothelial cells via an activation of NADPH oxidase, leading to superoxide generation and mitochondrial dysfunction, which ultimately cause endothelial dysfunction, inflammatory responses, and cell death [7]. This study demonstrates that UCP protected MGO-induced endothelial cell inflammation and apoptosis by suppressing ROS generation, prohibiting inflammatory signaling through iNOS, COX-2, and NF-κB, and modifying Akt-, p38-, and JNK-regulated apoptosis.

MGO-activated ROS generation is an early event in the reactive glycation and proteotoxic response to oxidative stress involving cellular inflammation and cell death [23]. In diabetes and other age-related diseases, the accumulated MGO glycates biomolecules, such as protein and DNA, leading to the formation of AGEs and the subsequent activation of RAGE expression. Circularly, MGO and its derivative hydroimidazolone-1 bind and activate RAGE to produce more ROS in human endothelial cells [24]. Therefore, suppression of ROS formation or cellular oxidative stress by phytoantioxidants is a crucial approach proven to prevent MGO-induced endothelial apoptosis. For instance, Pang et al. report that polydatin, a glucoside of resveratrol, prevents MGO-induced apoptosis in human umbilical endothelial cells (HUVECs) by inhibiting ROS formation and maintaining mitochondrial membrane potential [25]. Similarly, a study by Zhou et al. [26] indicates that the flavone apigenin inhibited AGE-induced oxidative stress and inflammatory signaling through ERK1/2 and NF-κB. This study shows that UCP attenuated intracellular ROS formation in endothelial cells at the basal condition, as well as in MGO-induced oxidative stress. The cytoprotective effect of UCP could be derived from its antioxidant core compositions, such as ascorbic acid, polyphenols, and flavonoids found in the fruit extracts [20,27].

The building up of ROS involves aging processes which can be accelerated by AGEs. MGO-derived AGEs triggered the RAGE cascade, leading to the activation of NADPH oxidase that generates O_2_^•−^ [28]. UCP mitigated intracellular O_2_^•−^ stress which plays a key role in NO-deprived endothelial dysfunction. Superoxide anion avidly reacts with NO in a bimolecular (1:1) diffusion-controlled reaction, and consequentially generates peroxynitrite (ONOO^−^) which further causes protein tyrosine nitration, DNA fragmentation, lipid peroxidation, and ultimately leads to mitochondria dysfunction and apoptosis [29]. The reaction of the overproduced O_2_^•−^ with NO critically shifts NO from physiological relaxant factor to a cytotoxic oxidant ONOO^−^ that modified a wide range of biomolecules, resulting in an accumulation of oxidized products that disturb cellular redox homeostasis and leaning the cells toward inflammatory responses and apoptosis [30]. For example, MGO-induced O_2_^•−^ generation corresponds to the impairment of NO-dependent vasorelaxation and enhanced levels of nitrotyrosine (the marker peroxynitrite), AGEs formation, and the inflammatory mediator monocyte chemoattractant protein-1 expression [31]. As a result, the action of UCP against oxidative stress is a crucial approach to prevent MGO-induced endothelial dysfunction and apoptosis.

eNOS is a key mediator in maintaining vascular tone and homeostasis by producing NO. In dicarbonyl stress condition, MGO causes endothelial dysfunction and cell damage by the uncoupling of eNOS and the destruction of NO [1]. The phosphorylation of eNOS was elevated in a short period of time at 1 h after MGO treatment, which may be associated with the activation of the upstream PI3K/Akt signaling [32]. An impairment of endothelial function was observed afterward at 4 h, where MGO caused a reduction in phospho-eNOS levels and an attenuation of NO release. Similar to a previous study in EA.hy926 cells, the phosphorylation of eNOS and NO production were decreased in MGO-exposed cells, leading to an increase in intracellular O_2_^•−^ production [33]. Surprisingly, total eNOS protein expression was decreased by UCP. It is possible that some phytochemicals in plant extracts act as NOS inhibitors such as curcumin and curcuminoid pyrazoles [34]. Thus, UCP might contain unknown NOS inhibitory compounds that should be further investigated. Although UCP inhibited eNOS protein expression, UCP did not change NO levels. It is likely that the antioxidant effect of UCP could maintain NO bioavailability by scavenging O_2_^•−^ and preventing ONOO^−^ generation [35].

MGO-induced a shift of cellular redox state toward oxidative stress causes detrimental effects exceeding endothelial dysfunction. ROS act as signaling molecules that activate MAPK (ERK, JNK, and p38), causing the translocation of the transcription factor NF-κB to the nucleus, which in turn, activate the transcription of a set of inflammatory mediator genes such as COX-2 and iNOS [4,5,36]. A pileup of glycolytic overload from MGO drives the development of metabolic diseases, such as obesity, atherosclerosis, diabetes mellitus, and aging, via the activation of NF-κB in endothelial cells [1]. Moreover, NF-κB induction contributes to endothelial dysfunction, increased production of proinflammatory cytokines, and insulin resistance in diabetes [37]. Our findings indicate that MGO significantly activated NF-κB (as early as 1 h), enhanced synthesis of NF-κB inflammatory response gene products, including COX-2 (at 2 h) and iNOS (at 6 h). UCP suppressed NF-κB signaling pathway and its downstream pro-inflammatory mediators. The proposed mechanisms involve the elimination of excessive intracellular ROS by its scavenging activities, along with its anti-inflammatory phytochemicals found in the fruit extracts such as chlorogenic acid, ellagic acid, and quercetin [38].

The process of ROS-induced cellular inflammation signifies a bridge between the oxidative stress and apoptosis. High concentrations of MGO invokes endothelial cell apoptosis via mitochondrial dysfunction, ROS/MAPK/NF-κB signaling pathway, and ER stress [39]. In endothelial cells, the redox-sensitive family members of MAPK triggered by MGO are ERK, JNK, and p38 MAPK [40]. We found that MGO induces endothelial cell apoptosis by promptly enhancing the phosphorylation of JNK and p38, but cells pretreated with UCP significantly decrease the MAPK phosphorylation and apoptotic cells. In addition to reducing the MAPK apoptotic signals, activation of PI3K/Akt is implicated in the endothelium protection against high glucose-induced apoptotic stress [41]. However, we found that MGO activated Akt phosphorylation at a very early time point (1 h) and this effect was attenuated by UCP. A chronic activation of Akt phosphorylation paradoxically causes cardiac cell hypertrophy in heart failure patients [42]. Therefore, the effect of UCP on the preservation of Akt phosphorylation levels in MGO treated cells may provide self-protection from the perturbation of PI3K/Akt signal triggered by oxidative stress.

Recent studies for the mechanisms of endothelial cytoprotection and anti-aging effects involve the activation of autophagy process. A study by Fang et al. [43] shows that autophagy inhibitors precipitated human brain microvascular endothelial cells apoptosis, which mimicked the endothelial cell injury observed in the model of permanent middle cerebral artery occlusion-induced cerebral ischemia in diabetic rats. Under metabolic stress, enhanced Akt phosphorylation is driven by AMPK that works in cooperation with SIRT1 to promote autophagy [44]. Although glucose starvation is a crucial factor to induce the activation of AMPK and SIRT1, our study showed that there were no significant changes in this signaling pathway, observed in both high and low glucose culture media. These data suggest that the underlying mechanisms of the protective effect of UCP against MGO-induced oxidative stress did not relate to the modification of autophagic activity, but primarily mediated by intracellular ROS reduction followed by an inactivation of the oxidative stress-sensitive MAPK signaling cascade (JNK and p38). Alternatively, UCP prevented inflammatory responses to AGEs/RAGE-related MGO stimulation.

In summary, the scavenging activity of UCP keeping the balance of excessive ROS and maintaining redox homeostasis are the key mechanisms to prevent endothelial cell inflammation and apoptosis from oxidative and dicarbonyl stress. Enormous generation of intracellular ROS from the mitochondria can induce ER stress and activate NADPH oxidase that subsequently drive endothelial cells toward inflammation and apoptosis by Akt/MAPK/NF-κB pathway. UCP protected endothelial cell inflammation and apoptosis by scavenging ROS and disrupting redox chain signaling cascades that activate NF-κB/iNOS/COX-2. Additionally, the scavenging activity of UCP can prevent NO from O_2_^•−^ attack, resulting in the retention of NO bioavailability. UCP reveals benefits of preventing endothelial inflammation and apoptosis under the MGO model of stimulated diabetic condition. Nevertheless, using UCP as a dietary supplement requires in vivo and clinical research to validate its action against endothelial-induced complications and aging in diabetes.

## 5. Conclusions

UCP protected MGO-induced endothelial cells oxidative stress by scavenging intracellular ROS production and inhibiting cellular inflammation and apoptosis via the deactivation of NF-κB/iNOS/COX-2 and Akt/JNK/p38 MAPK pathways. Thus, UCP shows considerable promise for developing as novel functional food and nutraceutical products to reduce risks of endothelial inflammation and vascular complications in diabetes.

## Data Availability

Data are contained within article.

## Supplementary Materials

The following are available online at https://www.mdpi.com/article/10.3390/antiox10081158/s1, Figure S1: Original image of Western blot band intensities in Figure 5; Figure S2: Original image of Western blot band intensities in Figure 6; Figure S3: Original image of Western blot band intensities in Figure 7; Figure S4: Original image of Western blot band intensities in Figure 8.
