# Supplementary material for: Unripe Carica papaya Protects Methylglyoxal-Invoked Endothelial Cell Inflammation and Apoptosis via the Suppression of Oxidative Stress and Akt/MAPK/NF-κB Signals
Source: Antioxidants (Basel). 2021 Jul 21;10(8):1158. doi: 10.3390/antiox10081158 (PMC8388906; doi:10.3390/antiox10081158)
Supplement: Supplementary file 1 [file antioxidants-10-01158-s001.zip › antioxidants-1284918-supplementary.pdf]

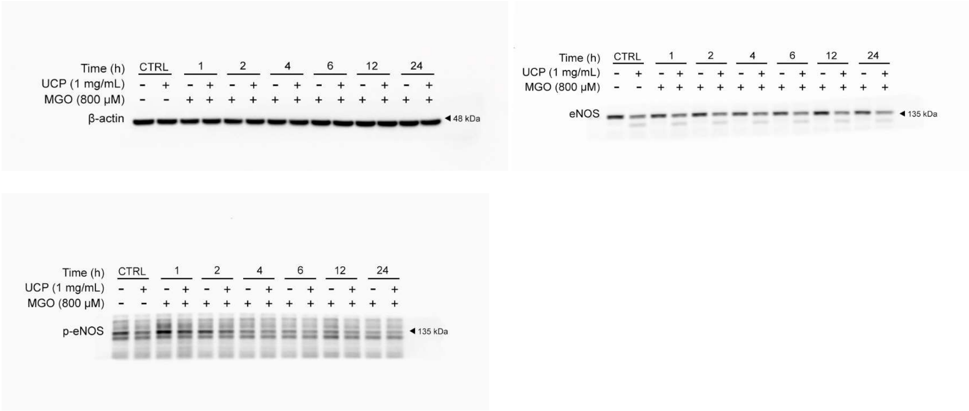

Figure S1 Original image of western blot band intensities in Figure 5

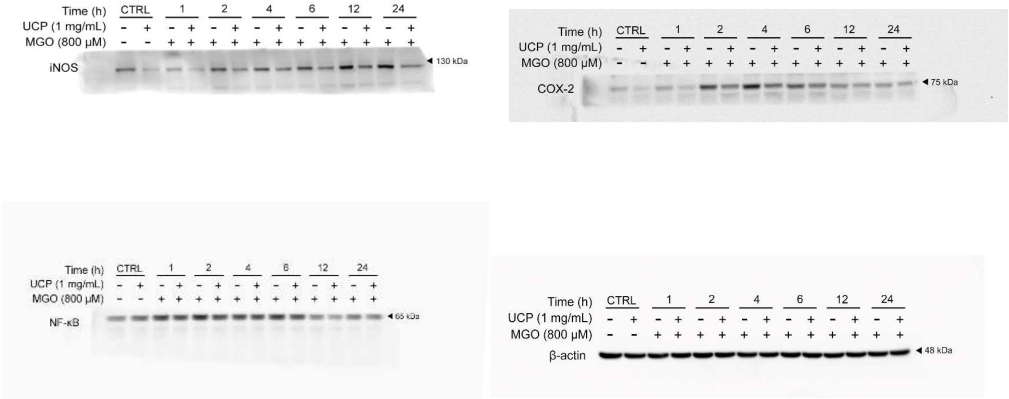

Figure S2 Original image of western blot band intensities in Figure 6

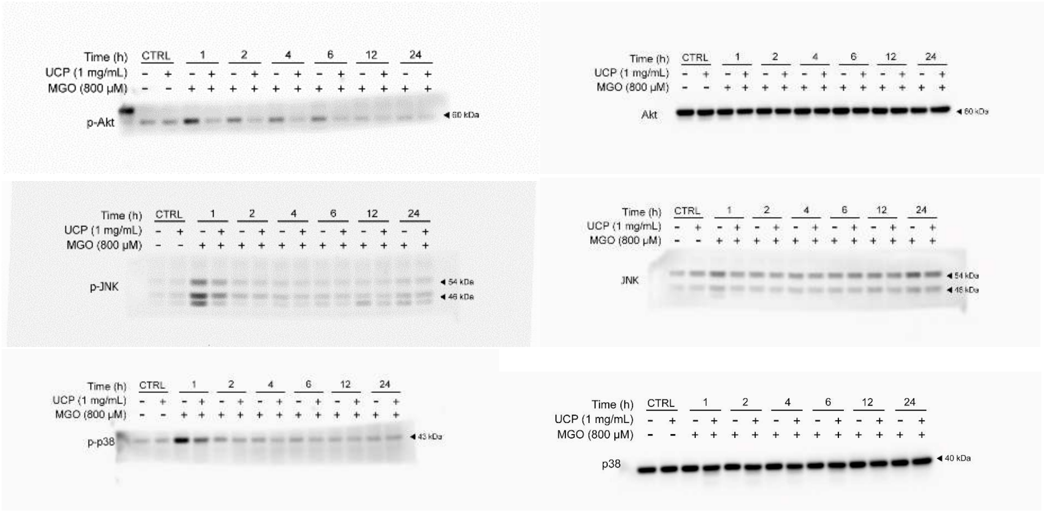

Figure S3 Original image of western blot band intensities in Figure 7

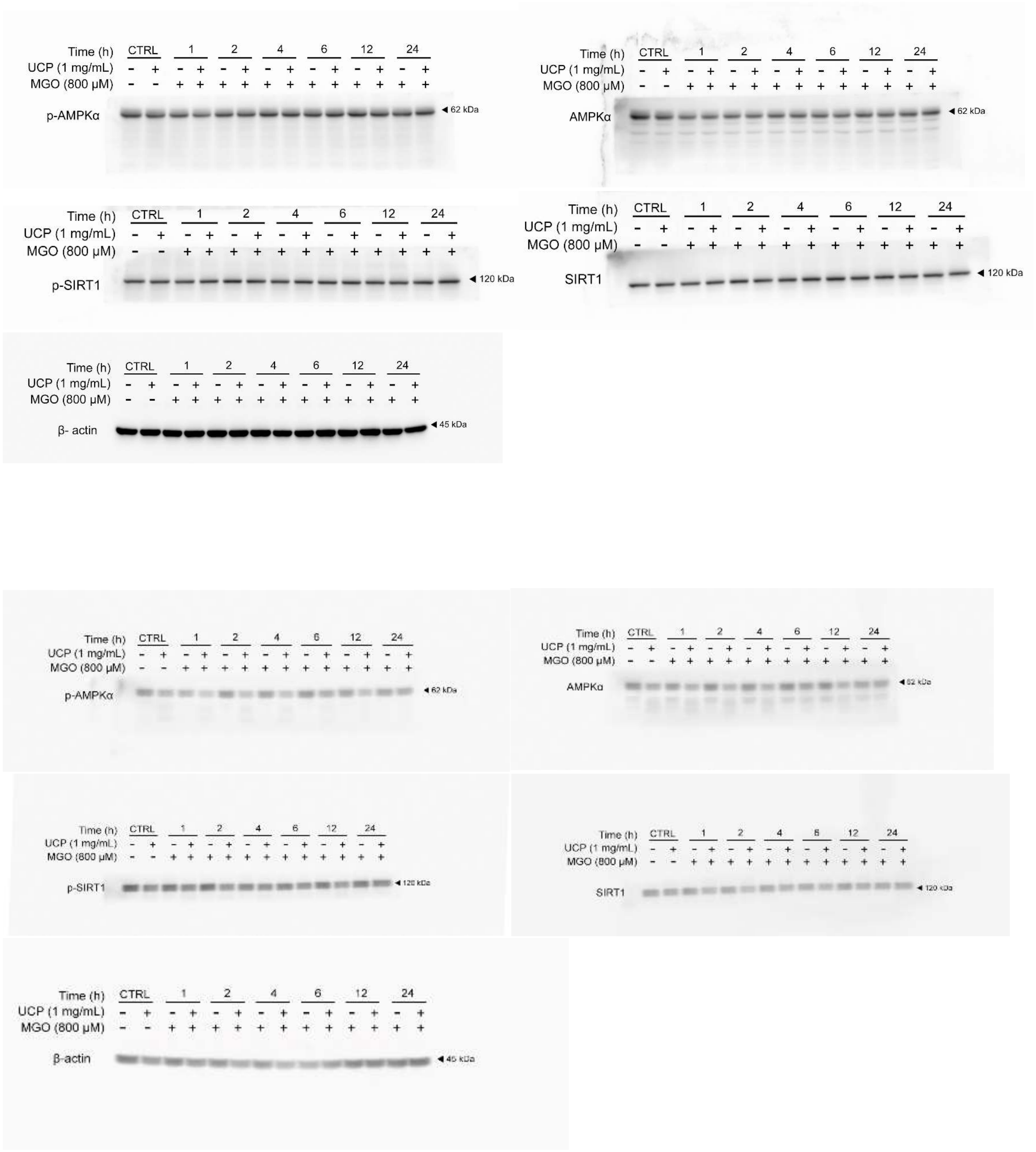

Figure S4 Original image of western blot band intensities in Figure 8
